# Complete mitochondrial genome of the feather mite *Ardeacarus ardeae* (Acari, Sarcoptiformes, Pterolichidae)

**DOI:** 10.1080/23802359.2017.1289345

**Published:** 2017-02-16

**Authors:** Yeong-Deok Han, Gi-Sik Min

**Affiliations:** Department of Biological Sciences, Inha University, Incheon, South Korea

**Keywords:** *Ardeacarus ardeae*, complete mitogenome, feather mite, Pterolichidae, Sarcoptiformes

## Abstract

In this study, we determined the mitogenome sequence of *Ardeacarus ardeae* (Canestrini, 1878) in the family Pterolichidae (Acari, Sarcoptiformes), which is the first complete mitogenome sequence in feather mite. The mitogenome of *A*. *ardeae* is 14,069 bp in length and contains 13 protein-coding genes (PCGs), 22 transfer RNAs (tRNAs), 2 ribosomal RNAs (rRNAs), and a control region (CR). The phylogenetic tree shows that *A*. *ardeae* belong to the supercohort Desmonomatides within the order Sarcoptiformes.

Feather mites are referred to as commensal or parasitic species that live on the plumage and skin of birds (Mironov 2003; Proctor 2003). *Ardeacarus ardeae* (Canestrini, 1878) (Acari, Acariformes, Sarcoptiformes, Pterolichidae), which is a monotypic species of the genus *Ardeacarus* Dubinin, 1951, has a worldwide distribution and is mostly observed on wing feathers of the birds in the family Ardeidae (Gaud 1981; Gaud & Atyeo 1996). Here, we present the complete mitogenome sequence of *A*. *ardeae* in family Pterolichidae, which is the first complete mitogenome from a feather mite.

A single specimen of *A*. *ardeae* was collected from a wing feathers of little egret *Egretta garzetta* collected from Eumseong-gun, Chungcheongbuk-do Province (36°56′N, 127°41′E). Mitochondrial DNA extraction, sequencing and gene annotation were performed according to the methods described by Song et al. (2016). The extracted mitochondrial DNA (Inhaevodevo-HYD-0030) was deposited in the Inha University, Incheon, South Korea. The phylogenetic tree was constructed based on the analysis of maximum likelihood (GTR + I + G model, PhyML 3.0) and Bayesian inference (GTR + I + G model, MrBayes 3.2.3) methods using the concatenated sequence of 13 protein-coding genes from the related 15 species within the superfamily Acariformes.

The complete mitogenome of *A*. *ardeae* (GenBank accession no. KY352304) was 14,069 bp in length and contained the typical set of 13 protein-coding genes (PCGs), 22 transfer RNAs (tRNAs), two ribosomal RNAs (rRNAs) and a control region (CR). The overall base composition of the entire *A*. *ardeae* mitogenome consisted of 29.3% A, 45.5% T, 10.8% C, 14.5% G and 74.8% AT. Twelve of the PCGs use an ATN codon (N, any nucleotide), while *atp*8 uses TTG. The stop codons of three PCGs are incomplete and use just one T nucleotide.

The gene arrangement of the *A*. *ardeae* mitogenome, with the exception of tRNAs, was found to be almost identical to those of previously published spemicies in Sarcoptiformes. However, *A*. *ardeae* has a shorter mitogenome than two closely related species, *Dermatophagoides farinae* (14,266 bp) and *D*. *pteronyssinus* (14,203 bp). These length differences are mainly due to the CR, which is 214 bp long in *A. ardeae*, while *D*. *farinae* and *D*. *pteronyssinus* have CRs composed of 410 and 286 bp, respectively (Dermauw et al. 2009; Klimov & Oconnor 2009).

We inferred the phylogenetic relationship of *A*. *ardeae* to the Acariformes species. The phylogenetic tree of maximum likelihood and Bayesian inference showed that *A*. *ardeae* belongs to the supercohort Desmonomatides within the order Sarcoptiformes. Furthermore, *A*. *ardeae* of the family Pterolichidae showed a sister-group relationship with the family Pyroglyphidae (*D*. *farinae* and *D*. *pteronyssinus*) (Figure 1). The phylogenetic relationships found in our study are well-congruent with previous mitogenome studies reported by Lee and Wang (2016) and Xue et al. (2016).

**Figure 1.**
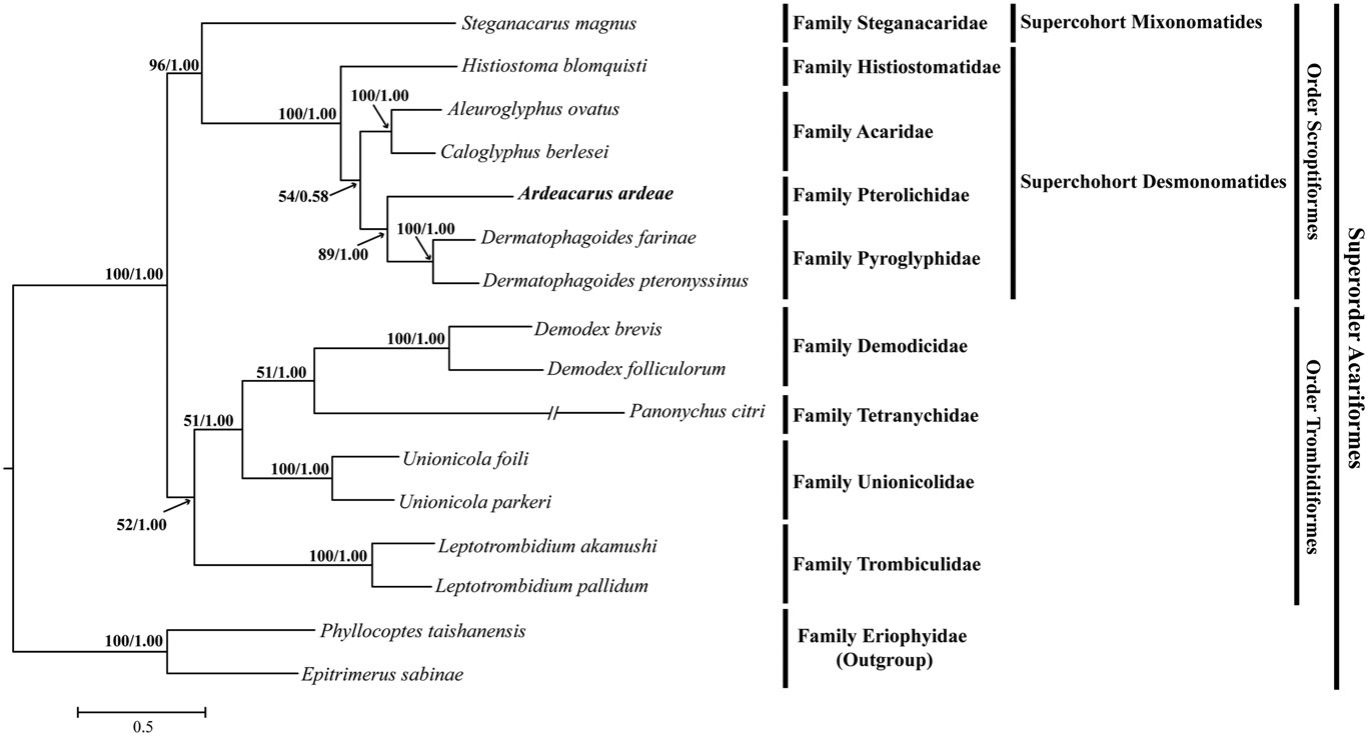
Maximum likelihood and Bayesian inference tree based on the mitogenome sequences of *Ardeacarus ardeae* (KY352304; this study) and 15 other Acariformes species was constructed using PhyML 3.0 and MrBayes 3.2.3, respectively. The following mitogenome were used in this analysis: *Aleuroglyphus ovatus* (KC700022), *Caloglyphus berlesei* (KF499016), *Demodex brevis* (KM114225), *D*. *folliculorum* (KM114226), *Dermatophagoides pteronyssinus* (EU884425), *D*. *farinae* (NC013184), *Histiostoma blomquisti* (KX452726), *Leptotrombidium pallidum* (AB180098), *L*. *akamushi* (AB194045), *Panonychus citri* (HM189212), *Steganacarus magnus* (EU935607), *Unionicola foili* (EU856396) and *U*. *parkeri* (HQ386015); outgroup: *Epitrimerus sabinae* (KR604966) and *Phyllocoptes taishanensis* (KR604967).

Our study determined the complete mitogenome of *A*. *ardeae* in the family Pterolichidae for the first time and will be useful for the detailed study of mitogenome evolution and phylogenetic relationships of the feather mite groups in the order Sarcoptiformes.
